# A systematic review and network meta‐analysis comparing Rezūm with transurethral needle ablation and microwave thermotherapy for the management of enlarged prostate

**DOI:** 10.1002/bco2.361

**Published:** 2024-04-29

**Authors:** Ansh Bhatia, Joao G. Porto, Renil S. Titus, Vishal Ila, Khushi Shah, Ankur Malpani, Diana M. Lopategui, Robert Marcovich, Thomas R. W. Herrmann, Hemendra N. Shah

**Affiliations:** ^1^ Desai Sethi Urology Institute, Miller School of Medicine University of Miami Miami Florida USA; ^2^ Seth GS Medical College and KEM Hospital Mumbai India; ^3^ Spital Thurgau AG Hospital Frauenfeld Switzerland

**Keywords:** benign prostatic hyperplasia, lower urinary tract symptoms, Rezūm, thermotherapy, transurethral microwave therapy, transurethral needle ablation, water vapour therapy

## Abstract

**Objectives:**

We aim to compare efficacy and safety of water vapour therapy (Rezūm), transurethral needle ablation (TUNA) and transurethral microwave therapy (TUMT) for treating men with moderate to severe benign prostatic hyperplasia (BPH) symptoms.

**Materials:**

PubMed/MEDLINE, EMBASE and Cochrane Library were searched from inception to 30 July 2023, followed by reference searching and dual‐independent study selection. We analysed only randomized clinical trials. RoB‐2, NIH‐quality assessment tool and GRADE guidelines were used for quality‐of‐evidence (QoE) assessment. Relevant prospective studies without a critical risk‐of‐bias were included.

**Results:**

At 12 months, Rezūm showed similar efficacy to TUNA and TUMT for improvement in International Prostate Symptoms Score – Rezūm versus TUMT: 1.33 points (95% CI: −1.66 to 4.35) favouring TUMT (QoE: Moderate) and Rezūm versus TUNA: 0.07 points (95% CI: −3.64 to 3.88) favouring TUNA (QoE: Low). Rezum had similar outcomes to TUNA and TUMT for Maximum Peak‐Flow Rate (Qmax): Rezūm versus TUMT: 1.05 mL/s (95% CI: −4.88 to 2.82) favouring Rezūm (QoE: Low) and Rezūm versus TUNA: 0.37 mL/s (95% CI: −4.61 to 4.21) favouring TUNA (QoE: Low). Furthermore, post‐void residual volume (PVR) comparisons demonstrated that Rezūm was similar, or inferior to other techniques at 12 months – Rezūm versus TUMT: 11.20 mL (95% CI: −32.40 to 10.30) favouring TUMT (QoE: Low) and Rezūm versus TUNA: 24.10 mL (95% CI: 2.81 to 45.10) favouring TUNA (QoE: Low). Rezūm also had a similar surgical retreatment rate with TUMT and TUNA up to 3‐years – TUMT versus Rezūm RR: 1.21 (95% CI: 0.20 to 15.90) (QoE: Low) and TUNA versus Rezūm showed RR: 1.81 (95% CI: 0.2 to 24.60) (QoE: Low). In the first 12 months after treatment, Rezūm had a higher rate of serious adverse events (Clavien‐Dindo ≥ Grade 3) than TUMT and TUNA. TUMT versus Rezūm with RR = 0.53 (95% CI: 0.13 to 3.14) (QoE: Low) and TUNA versus Rezūm with RR = 0.38 (95% CI: 0.04 to 3.49) (QoE: Low).

**Conclusions:**

Moderate to weak evidence suggests that Rezūm is not superior to TUNA and TUMT in all domains studied.

## 1 INTRODUCTION

Benign prostatic hyperplasia (BPH) is nearly ubiquitous amongst aging males, with prevalence increasing at age 40–45 and reaching 80% at age 80.^1^ For over a century after its initial description, transurethral resection of the prostate (TURP) has been the gold standard for surgical management of BPH.^2^ TURP effectively resects obstructive prostate tissue but is associated with potential morbidities.^3^ To mitigate the various drawbacks of TURP, various office‐based thermotherapy modalities have been introduced in the last few decades. Thermotherapy aims to heat the prostatic tissue to 45°C, which causes the targeted tissue to slough off over weeks to months.

The earliest evidence of clinical use of thermal therapy was in 1982 by Yerushalami et al., who treated a prostatic adenocarcinoma using microwave energy. The application was soon expanded to treat BPH patients who were poor surgical candidates.^4^ Microwaves with a frequency of 915–1296 MHz penetrate prostatic tissue and cause electromagnetic oscillations of free charges and polarization of water molecules. The kinetic energy released induces cell necrosis, vascular injury and apoptosis.^5^ In 1993, almost a decade after introducing transurethral microwave therapy (TUMT), a low‐level radiofrequency energy version of TUMT (460 kHz) was developed. It delivers heat directly to the prostate tissue, resulting in necrosis.^6^, ^7^ In 2004, NIH funded a study comparing the long‐term benefits and risks of transurethral needle ablation (TUNA) and TUMT to combined medical treatment.^8^ However, after more than 6 years, the study was terminated due to failure to recruit patients.

Both TUNA and TUMT, which employ conductive heat transfer, were popular in the 1990s–2000s and were included in the American Urological Association (AUA) BPH guidelines of 2003 and 2010 due to their minimally invasive nature and low adverse event rate.^9^, ^10^ The short‐lived efficacy and high retreatment rates (RTR) (> seven times that of TURP) of both methods^11^ eventually led to their decline in clinical practice and removal/restricted recommendations in the latest 2020 AUA guidelines.^12^ On the other hand, Rezūm is a recent addition to the thermotherapy armamentarium that uses convective heat transfer to ablate tissue. The radiofrequency energy is used to generate water vapour at 103°C, which is postulated to convectively disperse through the tissue interstices, where it shifts from vapour back to liquid. The energy released heats the prostate tissue to approximately 70°C, resulting in irreversible and instantaneous cell death.^13^ Rezūm has recently been included in the latest 2020 AUA BPH practice guidelines^9^ based on sham‐controlled and single‐arm study results.^12^ In contrast to the AUA guidelines, the 2022 European Urological Association (EAU) guidelines stress the need for randomized clinical trials (RCTs) comparing outcomes with Rezūm against a reference technique to confirm the efficacy, safety and durability of the procedure.^14^

To the best of our knowledge, while reviews exist for individual and pooled MIST comparisons,^11^, ^15^ no studies compare the outcome of various thermotherapies (TUNA, TUMT and Rezūm) specifically for treating BPH, especially at 1‐year outcomes. With this contextual backdrop, a pertinent question arises: Is Rezūm actually superior to its predecessors, TUNA and TUMT, or is it another heat‐based treatment with inferior benefit to current BPH techniques? To address this question, this network meta‐analysis (NMA) endeavours to assess the relative efficacy, safety and retreatment rates of Rezūm, TUMT and TUNA.

## 2 METHODS AND MATERIALS

This NMA was registered to the PROSPERO database (CRD42023397140) and was conducted following the PRISMA‐N guidelines.

### 2.1 Search strategy

A search of Medline, EMBASE and the Cochrane‐library database was conducted from inception to March 2023 (and re‐ran in July 2023 before the final analysis) with keywords (and combinations thereof) such as ‘Rezūm’, ‘Water vapor wave therapy’, ‘Transurethral Needle Ablation’, ‘Transurethral Microwave Therapy’ and ‘Benign Prostatic Hyperplasia’ with Boolean operators. The search strategy can be found in the supplemental dataset. Additional studies were searched for by going through the reference lists of the available articles.

### 2.2 Inclusion criteria and study selection

Prospective multi‐arm trials that compared the effect of Rezūm/TUMT/TUNA to TURP/Sham/TUNA/TUMT and had at least one of the following outcome variables: International Prostate Symptom Score (IPSS), Postvoid Residual Volume (PVR), Quality of Life (QoL), Maximum Peak Flow Rate (Qmax), retreatment and Serious Adverse Events (SAE) rates at one of the following time points: 3, 6, 12 and 24 months were included in the study. To comprehensively evaluate the available evidence while preserving the quality of evidence and foundations of evidence‐based medicine, two sets of quantitative analyses were conducted. One set only pooled RCT outcomes. The second set pooled included prospective single‐armed studies without a critical risk of bias.

**FIGURE 1:**
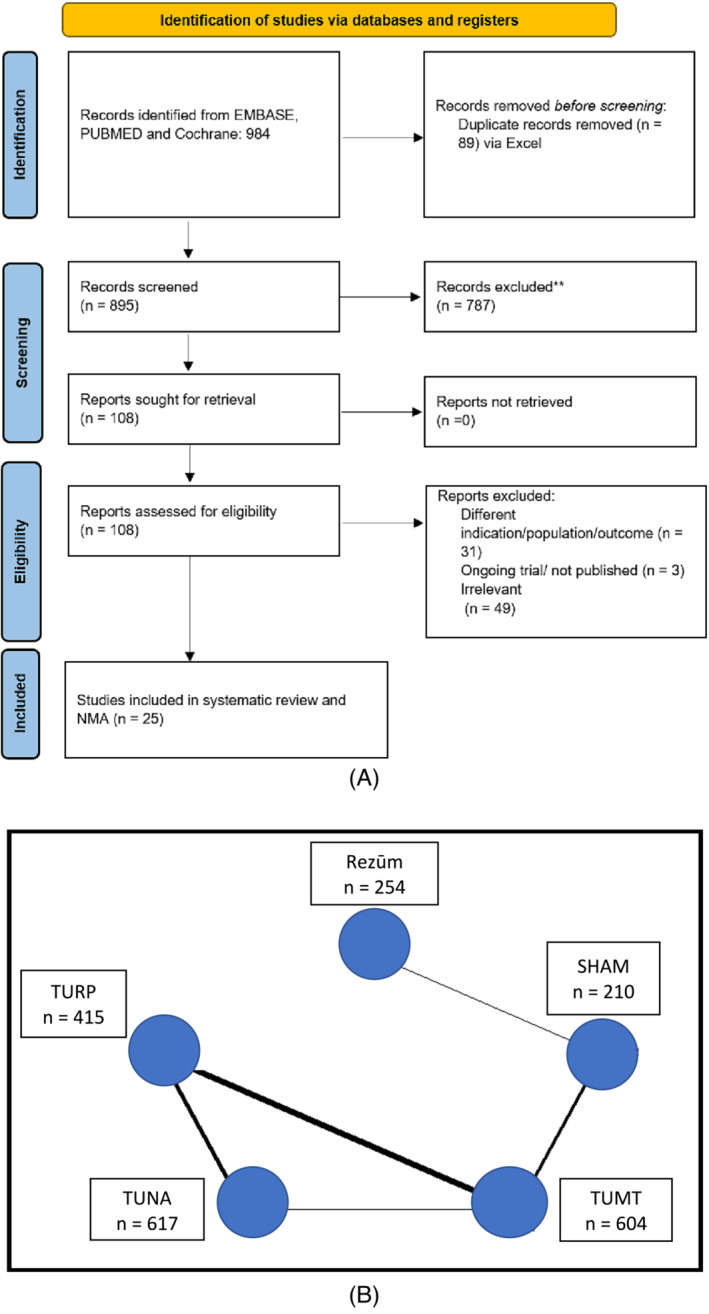
(A) Study selection process showing the assessment and reason for removal of studies at each stage. (B) Geometry of this network meta‐analysis.

**FIGURE 2:**
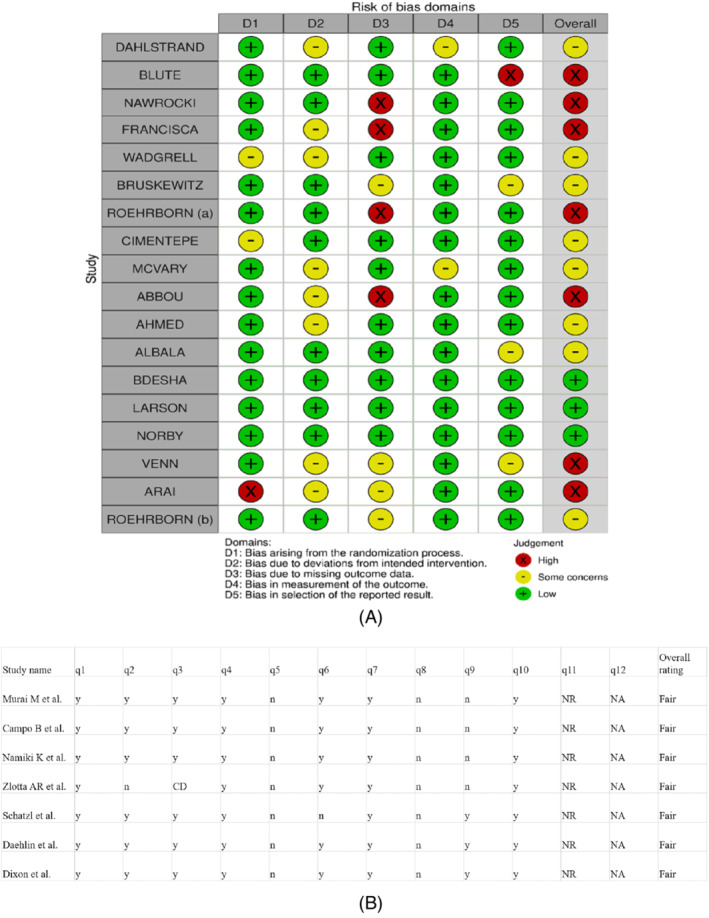
(A) Risk of Bias (RoB‐2) tool for randomized trials. (B) NIH quality assessment tool for Single‐arm studies.

We excluded studies that lacked the outcome variables being investigated, had different treatment indications, focused solely on cost analysis, or were case studies. The PICOS criteria were used to identify eligible studies, as shown in Table 1. Bipolar‐TURP and monopolar‐TURP were combined in the same node due to similar efficacy^16^ and the indirect nature of comparison in this study. The study selection process is depicted in Figure 1A. After the search results were exported to Excel, duplicates were removed via Excel commands. The studies were screened based on the title and abstract. Screened studies underwent full‐text review by three authors (A. B., R. T. and V. I.), and disagreement was resolved with consensus and input of a fourth member (J. G. P.). Three authors independently (R. T., A. B. and V. I.) extracted the data from the selected studies, and the extraction was cross verified. Data points for 3 and 12 months were utilized for quantitative synthesis of early postoperative and short‐term follow‐up. If the data for these time points were unavailable, the closest follow‐up data were used. The geometry of the network is represented in Figure 1B. Two authors (AB and RT) did a Risk of Bias Assessment using Cochrane's ROB‐2 assessment tool for RCTs and the NIH quality assessment tool for Non‐Randomized Studies to evaluate the studies' quality (Figure 2). The GRADE collaboration guidelines were used to assess the quality of the evidence.^17^ All discrepancies were resolved by consensus.

**TABLE 1:** Participants, interventions, comparators, intervention, outcomes (PICOs) table depicting the eligibility criteria of studies.

Participants	Intervention	Comparator	Outcomes	Description of outcome
Adult males with moderate to severe LUTS due to BPH	Rezūm	TURP*, TUNA, TUMT, SHAM	International Prostate Symptom Score (IPSS)	Assesses the quality of life or bother score based on the patient's perception of the problem. Scored from 0 to 35.
			Quality of Life Score (QoL)	Score determining the impact of BPH symptoms on the patient's quality of life. Scored from 0 to 6.
			Post‐void Residual volume (PVR)	Urodynamic metric that measures urine (mL) left in the bladder after an average micturition.
			Maximum urinary flow velocity (Q max)	Purely urodynamic metric that determines maximum urinary flow velocity achieved in micturition.
			Serious Adverse effects (Clavien‐Dindo ≥3)	Adverse effects that require surgical, radiological, or endoscopic interventions for correction.

### 2.3 Evidence synthesis

The data was analysed using RStudio Version 4.3.3 (RStudio, Boston, MA, USA). Bayesian modelling was used for this NMA. Continuous outcomes were analysed according to mean difference (MD) with 95% credible intervals (CI) to estimate the effect size. Categorical variables such as SAE and RTR were compared as risk ratios. An arm‐based NMA was performed to compare Rezūm with TUNA and TUMT, an approach used extensively in the literature using the RStudio package ‘pcnetmeta’^18^, ^19^, ^20^. ‘Netmeta’ and ‘meta’ packages were used to evaluate heterogeneity and publication bias as ‘pcnetmeta’ does not provide these functions.

Treatments were considered statistically superior if the 95% CI did not cross the line of no effect. Serious adverse events (SAE) (defined as ≥Clavien‐Dindo 3) and RTR were considered with the last available number of patients at the time of interest. NMA was performed separately for each outcome to determine the treatment effect estimate.

## 3 RESULTS

### 3.1 Quantitative analysis

**FIGURE 3:**
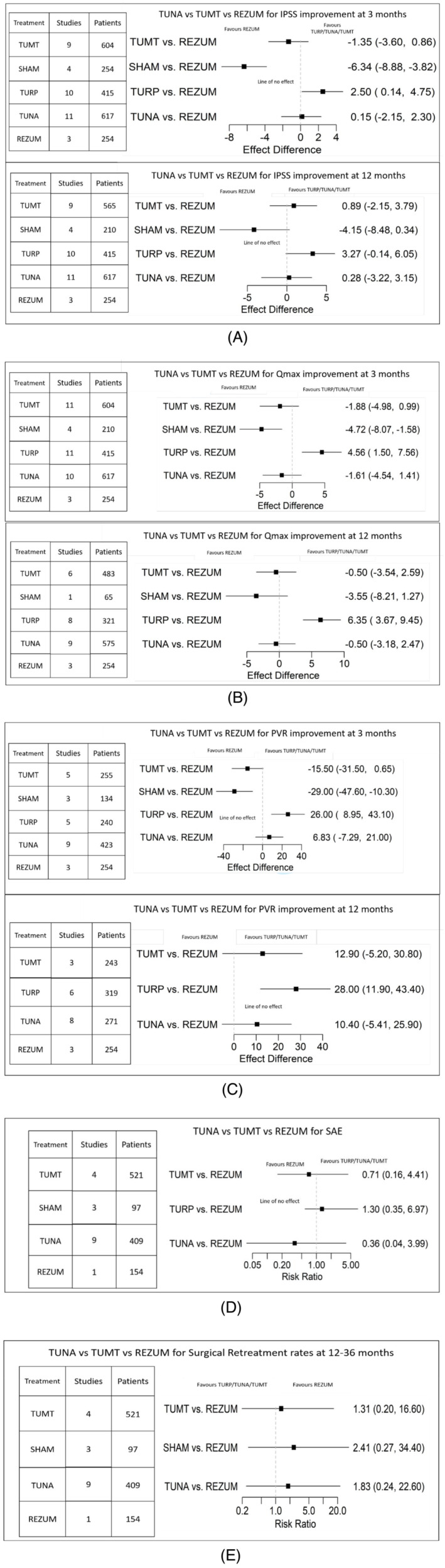
(A) IPSS changes at 3 and 12 months: TURP versus REZUM and SHAM versus REZUM are statistically significant for 3 months (above). None of the differences are statistically significant at 12 months (below). (B) Qmax comparisons at 12 months: TURP versus REZUM and SHAM versus REZUM are statistically significant for 3‐month comparison (above). TURP versus REZUM is statistically significant for 12 months (below). (C) Comparison of PVR 3 and 12 months: TURP versus REZUM and SHAM versus REZUM are statistically significant for the 3‐month comparison (above). TURP versus REZUM and SHAM versus REZUM is statistically significant for the 12‐month comparison (below). (D) Risk Ratios are statistically insignificant since all effect difference crosses line of no effect. (E) Risk Ratios are statistically insignificant since all effect difference crosses line of no effect.

Out of the 984 studies screened, 25^21^, ^22^, ^23^, ^24^, ^25^, ^26^, ^27^, ^28^, ^29^, ^30^, ^31^, ^32^, ^33^, ^34^, ^35^, ^36^, ^37^, ^38^, ^39^, ^40^, ^41^, ^42^, ^43^, ^44^, ^45^, ^46^ were incorporated into this NMA. The distribution of patients across treatments was as follows: TUMT with 604 patients, Rezūm with 254, TUNA with 617, Sham procedures with 210, and TURP with 415, culminating in 2100 patients. Analysis including only RCTs is presented in Table 2, while the analysis including single‐arm studies is shown in Figure 3. There was high heterogeneity in only the SAE and RTR outcomes. There was minimal within‐treatment, between‐study heterogeneity in the urodynamic and symptom scores. Funnel plots were used to assess publication bias in comparisons with more than 10 studies (Figure S1a–f).

**TABLE 2:** Outcomes of RCT only analysis.

Outcome	No. RCTs	Heterogeneity (*I* ^2^)*	Total patients included	Treatment	Effect size relative to REZUM	Quality of evidence (RCT only)
International Prostate Symptom Score (IPSS)						
3‐months	9	0%	658	TUMT	−0.607 (−3.460, 2.230)	Moderate^a^
	4	0%	274	SHAM	−5.330 (−8.260, −2.360)	Low^a^ ^,^ ^b^
	10	5.33%	454	TURP	3.140 (0.257, 6.030)	Moderate^a^
	6	0%	213	TUNA	0.499 (−2.450, 3.470)	Low^a^ ^,^ ^b^
12‐months	5	0%	367	TUMT	1.330 (−1.660, 4.350)	Moderate^a^
	2	0%	115	SHAM	−4.330 (−7.890, −0.621)	Moderate^a^
	5	0%	195	TURP	3.810 (0.750, 6.880)	Low^a^ ^,^ ^b^
	2	0%	41	TUNA	0.069 (−3.640, 3.880)	Low^a^ ^,^ ^b^
Maximum flow rate (Qmax)						
3‐months	10	0%	570	TUMT	−2.920 (−7.070, 1.380)	Moderate^a^
	4	0%	207	SHAM	−5.390 (−9.350, −1.260)	Low^a^ ^,^ ^b^
	10	0.93%	197	TURP	3.720 (−0.566, 8.300)	Moderate^a^
	4	55.16%	389	TUNA	−1.780 (−6.380, 3.150)	Very low^a^ ^,^ ^b^ ^,^ ^c^
12‐months	6	0%	373	TUMT	−1.050 (−4.880, 2.820)	Low^a^ ^,^ ^b^
	1	0%	65	SHAM	−3.980 (−9.330, 1.660)	Low^a^ ^,^ ^b^
	8	0%	321	TURP	5.910 (1.850, 9.720)	Moderate^a^ ^,^ ^b^
	3	57.80%	149	TUNA	−0.378 (−4.610, 4.210)	Low^a^ ^,^ ^b^ ^,^ ^d^
Post‐void residual volume (PVR)						
3‐months	4	0%	200	TUMT	−11.200 (−32.400, 10.300)	Low^a^ ^,^ ^b^
	3	0%	134	SHAM	−16.200 (−39.100, 6.960)	Low^a^ ^,^ ^b^
	5	0%	224	TURP	37.600 (16.500, 59.400)	Moderate^a^
	4	16.65%	147	TUNA	24.100 (2.810, 45.100)	Moderate^a^
12‐months	3	0%	191	TUMT	19.700 (−3.300, 42.900)	Low^a^ ^,^ ^b^
	6	54.76%	151	TURP	36.400 (15.500, 57.800)	Low^a^ ^,^ ^b^
	4	36.59%	241	TUNA	15.400 (−7.310, 37.900)	Low^a^ ^,^ ^b^
Retreatment rate						
	9	9%	436	TUMT	1.210 (0.198, 15.900)	Low^a^ ^,^ ^b^
	2	76%	31	SHAM	2.230 (0.247, 34.700)	Low^a^ ^,^ ^b^
	5	99.86%	300	TUNA	1.810 (0.285, 24.600)	Very low^a^ ^,^ ^b^ ^,^ ^c^
Serious adverse event						
	7	99.80%	437	TUMT	0.531 (0.127, 3.140)	Low^a^ ^,^ ^b^ ^,^ ^d^
	9	99.65%	498	TURP	1.350 (0.370, 7.230)	Very low^a^ ^,^ ^b^ ^,^ ^c^
	2	0%	130	TUNA	0.376 (0.038, 3.490)	Low^a^ ^,^ ^b^

*Note*: The asterisk (*) denotes within treatment. No study was downgraded due to publication bias as no significant bias was detected visually by funnel plots (Figure S7a–f) or Egger's test. Clinical heterogeneity (i.e., between‐trials differences in patients, treatments and outcomes characteristics) across eligible studies was assessed by examining details of participants and baseline characteristics. Statistical heterogeneity in individual pair‐wise meta‐analyses was explored with univariate tests for heterogeneity such as the estimates of the *I* ^2^ statistic. Statistical heterogeneity was considered moderate when *I* ^2^ = 50–74% and high when *I* ^2^ ≥ 75%.

Abbreviations: No., number; RCT, randomized clinical trial; TUMT, transurethral microwave therapy; TUNA, transurethral needle ablation; TURP, transurethral resection of the prostate.

^a^ Downgraded due within‐study risk of bias; most included studies had an elevated risk of bias.

^b^ Downgraded due to imprecision (wide confidence intervals).

^c^ Downgraded due to high heterogeneity.

^d^ Not downgraded further as imprecision is explained by the heterogeneity.

#### 3.1.1 International Prostate Symptom Score (IPSS)

In the RCT‐only comparison (Table 2), At the 3‐month mark, Rezūm demonstrated a significant advantage over Sham treatments in improving IPSS, with a difference of 5.33 points in favour of Rezūm (95% CI: −2.36 to −8.26, statistically significant). However, there were no statistically significant differences observed between Rezūm and TUMT (−0.61 points in favour of TUMT, 95% CI: −3.46 to 2.23) or between TUNA and Rezūm (0.50 points in favour of TUNA, 95% CI: −2.45 to 3.47). TURP, on the other hand, significantly outperformed Rezūm by 3.14 points (95% CI: 0.26 to 6.03); this difference was statistically and clinically significant (IPSS change >MCID of 3 points).^47^ The GRADE ratings for these findings can be seen in Table 2.

When including single‐arm studies, Rezūm significantly outperformed Sham at 3 months in IPSS improvement (6.34 points; 95% CI: 8.88 to 3.82, statistically significant). However, there was no statistically significant difference between Rezūm and TUMT (1.35 points in favour of Rezūm, 95% CI: −3.6 to 0.86) or between TUNA and Rezūm (0.15 points in favour of TUNA, 95% CI: −2.15 to 2.30). TURP significantly outperformed Rezūm (2.5 points favouring TURP, 95% CI: 0.14 to 4.75, statistically significant) (Figure 3A). The GRADE rating for these comparisons was uniformly ‘very low’ due to the inclusion of non‐randomized studies, risk‐of‐bias and substantial imprecision.

In the RCT‐only analysis at 12 months, Rezūm did exhibit a statistically significant improvement over Sham treatments in IPSS, with a difference of 4.33 points in favour of Rezūm (95% CI: −7.89 to −0.62). Similarly, there were no statistically significant differences between Rezūm and TUMT (1.33 points in favour of TUMT, 95% CI: −1.66 to 4.35) or between TUNA and Rezūm (0.07 points in favour of TUNA, 95% CI: −3.64 to 3.88). TURP, however, continued to significantly outperform Rezūm by 3.81 points (95% CI: 0.75 to 6.88), and this difference was statistically and clinically significant (Figure 3A). The GRADE ratings for each intervention can be seen in Table 2. The GRADE rating for these comparisons was uniformly ‘very low’ due to the inclusion of non‐randomized studies, risk‐of‐bias and substantial imprecision.

When including single‐arm trials, At 12 months, Rezūm outperformed Sham treatments in IPSS improvement (4.15 points in favour of Rezūm; 95% CI: −8.48 to 0.34), but this difference was statistically insignificant. There was no statistically significant difference between Rezūm and TUMT (0.89 points in favour of TUMT, 95% CI: −2.15 to 3.79) or between TUNA and Rezūm (0.28 points in favour of TUNA, 95% CI: −3.22 to 3.15). TURP outperformed Rezūm (3.27 points favouring TURP, 95% CI: −0.14 to 6.05) (Figure 3A). The GRADE rating for these comparisons was uniformly ‘very low’ due to the inclusion of non‐randomized studies, risk‐of‐bias and substantial imprecision.

#### 3.1.2 Maximum urinary flow rate (Qmax)

RCT only: At the 3‐month assessment, Rezūm did exhibit a statistically significant difference compared with Sham treatments in Qmax, with a difference of −5.39 mL/s in favour of Rezūm (95% CI: −9.35 to −1.26). There were no statistically significant differences between Rezūm and TUMT (−2.92 mL/s in favour of TUMT, 95% CI: −7.07 to 1.38) or between TUNA and Rezūm (−1.78 mL/s in favour of TUNA, 95% CI: −6.38 to 3.15). TURP outperformed Rezūm by 3.72 mL/s (95% CI: −0.57 to 8.30), but this difference was not statistically significant. The GRADE rating for the comparison can be seen in Table 2.

When including single‐arm studies: At 3 months, Rezūm significantly outperformed Sham in Qmax improvement (4.72 mL/s in favour of Rezūm; 95% CI: −8.07 to −1.58, statistically significant). At the same time point, there was no statistically significant difference between Rezūm and TUMT (1.88 mL/s in favour of Rezūm, 95% CI: −4.98 to 0.99) or between TUNA and Rezūm (1.61 mL/s in favour of Rezūm, 95% CI: −4.54 to 1.41). TURP significantly outperformed Rezūm (4.56 mL/s in favour of TURP, 95% CI: 1.50 to 7.56, statistically significant) (Figure 3B). The GRADE rating for these comparisons was uniformly ‘very low’ due to the inclusion of non‐randomized studies, risk‐of‐bias and substantial imprecision.

RCT only: At 12 months: Rezūm did not demonstrate a statistically significant difference compared with Sham treatments in Qmax, with a difference of −3.98 mL/s in favour of Rezūm (95% CI: −9.33 to 1.66). Similarly, there were no statistically significant differences between Rezūm and TUMT (−1.05 mL/s in favour of TUMT, 95% CI: −4.88 to 2.82) or between TUNA and Rezūm (−0.38 mL/s in favour of TUNA, 95% CI: −4.61 to 4.21). Conversely, TURP clinically (MCID of 2 mL/s) and statistically significantly outperformed Rezūm by 5.91 mL/s (95% CI: 1.85 to 9.72). The GRADE rating for the comparison can be seen in Table 2.

At 12 months, there was no statistically significant difference in Qmax improvement between Rezūm and Sham (3.55 mL/s in favour of Rezūm; 95% CI: −8.21 to 1.27). At the same time point, there was no statistically significant difference between Rezūm and TUMT (0.50 mL/s in favour of Rezūm, 95% CI: −3.54 to 2.59) or between TUNA and Rezūm (0.50 mL/s in favour of Rezūm, 95% CI: −3.18 to 2.47). TURP clinically (MCID of 2 mL/s) and statistically significantly outperformed Rezūm (6.35 mL/s in favour of TURP, 95% CI: 3.67 to 9.45, statistically significant) (Figure 3B). The GRADE rating for these comparisons was uniformly ‘very low’ due to the inclusion of non‐randomized studies and substantial imprecision.

#### 3.1.3 Post‐void residual volume (PVR)

In the RCT‐only analysis at the 3‐month assessment, Rezūm did not exhibit a statistically significant difference compared with Sham treatments in PVR, with a difference of −16.20 mL in favour of Rezūm (95% CI: −39.10 to 6.96). Similarly, there were no statistically significant differences between Rezūm and TUMT (−11.20 mL in favour of TUMT, 95% CI: −32.40 to 10.30). TUNA outperformed Rezūm by 24.10 mL (95% CI: 2.81 to 45.10, statistically significant). TURP also significantly outperformed Rezūm by 37.60 mL (95% CI: 16.50 to 59.40), which was statistically significant. The GRADE rating for the comparison can be seen in Table 2.

When including single‐arm studies at 3 months, Rezūm significantly outperformed Sham in PVR improvement (29 mL in favour of Rezūm; 95% CI: −47.6 to −10.30, statistically significant). At the same time point, there was no statistically significant difference between Rezūm and TUMT (15.50 mL in favour of Rezūm, 95% CI: −31.50 to 0.65) or between TUNA and Rezūm (6.83 mL in favour of TUNA, 95% CI: −7.29 to 21.00). TURP significantly outperformed Rezūm (26.0 mL in favour of TURP, 95% CI: 8.95 to 43.10, statistically significant). The GRADE rating for these comparisons was uniformly ‘very low’ due to the inclusion of non‐randomized studies and substantial imprecision.

In the RCT‐only analysis at the 12‐month assessment, Rezūm did not exhibit a statistically significant difference compared with TUMT in PVR, with a difference of 19.70 mL in favour of Rezūm (95% CI: −3.30 to 42.90). Similarly, there were no statistically significant differences between TUNA and Rezūm (15.40 mL in favour of TUNA, 95% CI: −7.31 to 37.90). However, TURP significantly outperformed Rezūm by 36.40 mL (95% CI: 15.50 to 57.80), which was statistically significant. The GRADE rating for the comparison can be seen in Table 2.

When including single‐arm studies, at 12 months, there was no statistically significant difference in PVR improvement between Rezūm and TUMT (12.90 mL in favour of TUMT. 95% CI: −5.20 to 30.80) or between Rezūm and TUNA (10.40 mL in favour of TUNA, 95% CI: −5.41 to 25.90). TURP significantly outperformed Rezūm (28 mL in favour of TURP, 95% CI: 11.90 to 43.40). No study reported Sham outcomes for PVR at 12 months. The GRADE rating for these comparisons was uniformly ‘very low’ due to the inclusion of non‐randomized studies and substantial imprecision.

#### 3.1.4 Serious adverse events (SAE)

In the RCT‐only analysis, TUMT had a lower Serious Adverse Events (SAE) rate than Rezūm; however, the difference was not statistically significant (RR = 0.53, indicating TUMT has a lower SAE rate; 95% CI: 0.13 to 3.14). Similarly, TUNA had a lower but statistically insignificant SAE rate than Rezūm (RR = 0.38, indicating that TUNA has a lower SAE rate, with 95% CI; 0.04 to 3.49). TURP had a higher SAE rate than Rezūm, and the difference was not statistically significant (RR = 1.35, indicating TURP has a higher SAE rate, with 95% CI: 0.37 to 7.23). The GRADE rating for the comparison can be seen in Table 2. There was significant heterogeneity in these results; we believe this is likely because of differential reporting and that some studies did not explicitly mention the Clavien–Dindo grade of their reported events. This resulted in the authors having to assign the relevant grade from the description of each adverse event described in the result/discussion.

When including the SAE rates from single‐arm studies, TUMT had a lower SAE rate than Rezūm; however, the difference was not statistically significant. (RR = 0.71, indicating TUMT has a lower SAE rate; 95% CI: 0.16 to 4.41). Similarly, TUNA had a lower but statistically insignificant SAE rate than Rezūm (RR = 0.36, indicating that TUNA has a lower SAE rate, with 95% CI; 0.04 to 3.99). TURP had a higher SAE rate than Rezūm, and the difference was not statistically significant (RR = 1.30, indicating TURP has a higher SAE rate, with 95% CI: 0.36 to 6.95). A comparison for SAE rates for the Sham comparison could not be performed because most studies report 0 events for the Sham group. The GRADE rating for these comparisons was uniformly ‘very low’ due to the inclusion of non‐randomized studies and substantial imprecision.

#### 3.1.5 Surgical retreatment rates (RTR)

Surgical retreatment was defined as treatment failure requiring repeat intervention for symptom improvement within 36 months. In the RCT‐only analysis, the surgical retreatment rate at the 12‐month assessment showed varying results across treatments. TUMT had a higher surgical retreatment rate than Rezūm (RR = 1.21, 95% CI: 0.20 to 15.90). Similarly, TUNA had a higher RTR than Rezūm, although the difference was not statistically significant (RR = 1.81, indicating lower RTR for Rezūm, with 95% CI; 0.2 to 24.60). Interestingly, although patients undergoing Sham had higher RTR, there was no statistically significant difference between Sham and Rezūm (RR = 2.23, indicating lower RTR with Rezūm, with 95% CI: 0.25 to 34.47). The GRADE rating for these comparisons can be seen in Table 2. There was significant heterogeneity in these results; we believe this is likely because of differential reporting, and some studies did not explicitly classify the reason for retreatment. This resulted in the authors having to infer the reason for retreatment described in the result/discussion of the corresponding publication. This heterogeneity also likely contributed to the wide confidence intervals obtained.

When including single‐arm studies, TUMT had a higher RTR than Rezūm, and the difference was not statistically significant (RR = 1.31, indicating lower RTR for Rezūm; 95% CI: 0.20 to 16.60). Similarly, TUNA had a higher RTR than Rezūm, although the difference was not statistically significant (RR = 1.83, indicating lower RTR for Rezūm, with 95% CI; 0.24 to 22.60). Interestingly, although patients undergoing Sham had higher RTR, there was no statistically significant difference between Sham and Rezūm (RR = 2.41, indicating lower RTR with Rezūm, with 95% CI: 0.27 to 34.40). A direct comparison of RTR between TURP and Rezūm was not feasible, as most studies indicated zero retreatment instances in the TURP group. The GRADE rating for these comparisons was uniformly ‘very low’ due to the inclusion of non‐randomized studies and substantial imprecision.

### 3.2 Qualitative analysis

The quality of evidence ranged from moderate to very low, according to the GRADE criteria.^48^ Most studies had at least a ‘somewhat significant’ risk of bias (Figure 2). It should be noted that most studies had bias in the concealment dimension of the RoB analysis, but concealment is not feasible for most surgical interventions, especially when the procedure under evaluation are MISTs, which do need general anaesthesia.

Broad geographic and temporal trends could be seen in some functional outcomes. Most TUNA and TUMT studies were from Europe, while Rezūm studies were mainly based in North America. The latest follow‐ups of patient cohorts describing TUNA, TUMT and Rezūm ended in 2004, 2005 and 2022, respectively.^27^ Given the strict exclusion criteria of Rezūm trials,^27^ the study population is unlikely to represent the target population, given real‐life comorbidities in a community setting. Real‐world outcomes align closely with the results from the pivotal Rezūm trial; however, they are consistently marred by insufficient reporting.^49^

The limited quality of reporting in TUMT and TUNA studies restricts the conclusions that can be made about their sexual impact and catheterization outcomes in comparison to Rezūm. (Table S1). This is partly because of the lack of availability of some objective tools/scoring systems like the SHIM/IIEF‐5 scales when the TUNA/TUMT studies were published. The available evidence suggests that TUNA, TUMT and Rezūm did not significantly impact sexual and ejaculatory function.^35^, ^50^

Catheterization outcomes are varied after TUNA and TUMT. Twenty studies reported either initial catheterization rates/time or re‐catheterization rate/time (defined as needing re‐insertion of Foley's catheter for urinary evacuation after prior successful voluntary voiding). The protocols regarding catheterization after TUMT/TUNA/Rezūm varied from no catheterization unless indicated (such as acute urinary retention, overflow incontinence, bladder irrigation) to catheterizing all patients regardless of indication after heat‐based therapy (ranging from 1 to 14 days). Overall, the evidence regarding catheterization and re‐catheterization is highly variable and poorly reported for TUNA and TUMT. All Rezūm studies indicated catheterization of all patients for at least 3–7 days unless contraindicated (Table S2). One study reported that Rezūm could be used to treat catheter‐dependent incontinence, resulting in 100% of patients being catheter‐free months after the procedure.^51^

Nine studies mention PSA, but most provide this data only at baseline (Table S3). These studies utilized PSA as a criterion to include or exclude patients for screening. No notable trends in serum PSA levels were observed after heat‐based procedures, as most studies did not report follow‐up PSA levels. Similarly, Prostate Volumes (PV) have been primarily reported at baseline, with most studies using different prostate volumes as a criterion for inclusion and exclusion. Most studies include 30–80 g PVs (Table S4).

## 4 DISCUSSION

This NMA showed Rezūm had similar voiding outcomes, SAE and RTR to TUNA and TUMT. Rezūm had inferior outcomes to TURP and similar SAE rates in the same domains. Additionally, at 3 and 12 months, Rezūm, TUNA and TUMT exhibited similar improvements in IPSS from their baseline values. It should be noted that the inclusion of large single‐arm studies did not substantially change the outcomes compared with the RCT‐only analysis. The sample sizes increased, although including single‐arm studies degraded the quality of evidence for each comparison.

However, TURP outperformed the heat‐based therapies at both time points, echoing findings from other meta‐analyses.^7^, ^11^ Limited long‐term data indicates that IPSS scores typically increase 2 years post‐thermotherapy, whereas after TURP, the rise in IPSS scores is slower.^52^, ^53^ At the 3‐ and 12‐month marks, Qmax outcomes for all heat‐based therapies were consistent yet notably lower than those for TURP. Other MISTs have reported Qmax improvements comparable to TURP.^15^ PVR outcomes were similar for Rezūm, TUNA and TUMT. TURP performed significantly better than Rezūm. The absolute difference between TURP and Rezūm was 25 mL; whether this difference is clinically relevant is debatable. One caveat to the PVR outcome comparison is the different PVR baseline values in various studies. These baselines were more heterogeneous than Qmax and IPSS baselines.

The difference in SAE rates of Rezūm, TUNA and TUMT was not statistically significant. While TURP had a higher SAE rate than Rezūm (RR: 1.3), the difference was not statistically significant. This result is likely due to the indirect nature of this analysis and the inherent uncertainty associated with the assumed transitivity of NMAs. Real‐world effect differences are likely significant, with TURP having higher SAE rates.^11^ Interestingly, TUMT and TUNA had lower SAE rates than Rezūm (RR 0.71 and 0.36, respectively). Based on our findings, when these lower SAE rates are viewed in conjunction with similar efficacy outcomes in symptom and urodynamic scoring, justifying Rezūm over TUNA and/or TUMT would be difficult.

While the authors of the pivotal Rezūm trial claim a surgical RTR of 4.4%,^27^ this includes all patients at baseline, not those lost to follow‐up. For a standard comparison, the retreatment rate calculation for this NMA used the last available follow‐up sample size, with uniform application across the TUNA, TUMT and Rezūm studies. This concern about Rezūm's RTR is hardly new, having been highlighted in an editorial shortly after the publication of the pivotal Rezūm trial.^54^ This NMA provides the first statistical evidence that the RTR does not significantly differ from other heat‐based therapies. Another recent study that evaluated real‐world outcomes based on registry‐level data reported that Rezūm has a retreatment rate of approximately 12%,^55^ almost three times the rate reported in the discussion of the cross‐over Rezūm trial.^27^

Surprisingly, two recent prospective studies detailing real‐world outcomes after Rezūm have reported retreatment outcomes without adhering to standard practice.^49^, ^56^ These studies do not provide the total number of patients at follow‐up time points and use only the baseline sample size to calculate the RTR. Multiple Rezūm studies and author groups also do not consider continued/restarting medical therapy for BPH as retreatment.^27^, ^54^ One recent study of real‐world outcomes reported that up to 45% of patients with prostate sizes between 30 and 50 g needed medical retreatment with alpha‐blockers in the 1 year following Rezūm.^57^, ^58^ In prostates sized 50–80 g, 50% of patients required retreatment with alpha‐blockers.^58^ While less cumbersome (and less effective) than surgical retreatment, medical retreatment can still significantly contribute to healthcare costs^57^, ^59^, ^60^ and should be considered when deliberating and reviewing treatment choices.

A recent argument for MISTs such as Rezūm is that retreatment rates and long‐term efficacy should not be critical considerations as these procedures can and should be repeated every 12–18 months due to their minimally invasive nature.^61^ For the efficacy provided by Rezūm, this is not a sustainable solution due to the cost of each repeat procedure and patients' difficulty accessing care repeatedly. Moreover, repeat treatments' urodynamic efficacy and safety effects remain to be investigated.

An additional advantage prominently highlighted to both urologists and the general public is Rezūm's ejaculatory‐sparing feature.^58^ it is essential to note that a substantial 70% of BPH patients may already exhibit symptoms of ejaculatory or erectile dysfunction before pursuing treatment. Surprisingly, only 35% of these patients perceive ejaculatory disorders as a substantial concern,^62^ with the majority attributing greater importance to the impact of lower urinary tract symptoms on their quality of life.

This situation prompts a critical inquiry: Does the potential reduction in treatment efficacy justify preserving ejaculatory function? This necessitates comprehensive discussions with patients during the initial goal‐setting phase and when selecting the most suitable treatment approach within a shared decision‐making framework. Managing patient expectations regarding treatment outcomes becomes paramount and should be a central consideration when recommending the optimal treatment strategy for each individual.

The results of this NMA suggest that Rezūm fails to demonstrate superiority over TUNA and TUMT, treatments whose usage has declined in recent years. In echoes of the early 2000s, Rezūm was included in the current AUA guidelines based on a single cohort of sham‐controlled patients and multiple publications on that same cohort. TUNA and TUMT were also initially included^9^ based on case series and sham‐controlled trials until comparison with TURP and long‐term follow‐ups showed an unacceptably high retreatment rate. Currently, the only available data longer than 3 years is from the original sham‐controlled trial.^27^ The AUA guideline 2021 rated the recommendations for Rezūm use as moderate, suggesting a moderate net benefit over harm, and as conditional, implying no discernible net benefit or harm, all founded on the ‘C' strength of evidence, which is categorized as low.^12^ Notably, the EAU does not recommend Rezūm and maintains that comparisons with an established technique, such as TURP, are needed before a final recommendation.^14^

Speakman et al.^63^ have proposed a comprehensive research roadmap for assessing new BPH treatments. They suggest that any new procedure should start with initial single‐arm human studies, followed by RCT comparisons with Sham procedures to evaluate safety. After this, a direct comparison with established methods like TURP or other accepted interventions is necessary to determine the true role of the newer technique in patient management. Given the strict inclusion and exclusion criteria in most initial studies (Table S5), the authors emphasized the importance of establishing the generalizability of results across diverse patient populations. As it stands, Rezūm is being widely adopted in clinical practice, even though it currently occupies a relatively lower tier within the evidence hierarchy. Further investigation may eventually yield to the emergence of more robust, high‐quality data.

This study has several limitations. The indirect comparisons may not reflect real‐world‐head comparisons between Rezūm, TUMT, TUNA and TURP. Since both TUNA and TUMT are essentially superseded by other MIST procedures, this comparison might be largely a historical curiosity rather than one that will inform a contemporary urologist about management decisions. Moreover, innovations in the Rezūm technique/device may improve outcomes, which may better justify Rezūm as a BPH treatment option. Another limitation is the poor reporting and lack of specific metrics (like the IPSS‐QoL) in most TUNA and TUMT studies. The analysis also does not examine outcome measures such as quality of life and sexual function that may motivate men to choose REZUM over traditional BPH surgeries. Additionally, only three studies including 254 patients were part of Rezum analysis. However, we would like to reinforce that the reason for this small sample size is that combining different study designs, such as RCTs and prospective non‐comparative single arm trials is not considered a valid strategy. Therefore, we have compared only RCTs. Additionally, we have included single arm studies separately in this analysis in order to preserve the principles of evidence‐based medicine. Despite this limitation based on the outcomes of this NMA, Rezūm has not been shown to substantially outperform older therapies and the widespread use of Rezūm is based on Sham‐controlled studies. Future research will benefit from comparing Rezūm with a standard surgical modality to evaluate its true relative efficacy instead of relying on sham‐controlled trials.

## 5 CONCLUSION

The widespread use of Rezūm in clinical practice is based on limited evidence, and the evidence suggests that Rezūm is not significantly better than TUNA and TUMT in any clinically relevant domain.

## AUTHOR CONTRIBUTIONS

**Ansh Bhatia:** Methodology; data collection; formal analysis and investigation; writing—original draft preparation; writing—review and editing. Joao **Gabriel Porto:** Data collecton; writing—review and editing. **Renil S. Titus:** Data collection; methodology; formal analysis; investigation; writing—original draft preparation. **Vishal Ila Langade:** Methodology; formal analysis; investigation. **Khushi Shah:** Data collection; methodology; writing—review and editing. **Ankur Malpani:** Data collection; writing—review and editing. **Diana M. Lopategui:** Data collection; writing—review and editing. **Robert Marcovich:** writing—review and editing; supervision. **Thomas R. W. Herrmann:** writing—review and editing; supervision. **Hemendra Navinchandra Shah:** Methodology; writing—review and editing; supervision.

## CONFLICT OF INTEREST STATEMENT

None of the authors has any conflicts of interest to declare.

## Supporting information


**Table S1.** Studies reporting Erectile dysfunction after TUMT/TUNA/REZUM/TURP/SHAM
**Table S2.** Comparison of Catheterization and re‐catheterization outcomes after TUNA, TUMT, REZUM studies.
**Table S3.** PSA levels at baseline. The remaining studies have not reported PSA levels at any time point.
**Table S4.** Studies describing the Prostate Volumes at baseline and 3–6 months.
**Table S5.** The different types of devices and techniques used may account for heterogeneous peri‐operative outcomes and their inclusion and exclusion criteria.

**Figure S1.** Funnel plot for assessing publications bias in IPSS at 3 months.
