# Supplementary material for: A systematic review and network meta‐analysis comparing Rezūm with transurethral needle ablation and microwave thermotherapy for the management of enlarged prostate
Source: BJUI Compass. 2024 Apr 29;5(7):621–35. doi: 10.1002/bco2.361 (PMC11250421; doi:10.1002/bco2.361)
Supplement: Supplementary file 1 — Table S1. Studies reporting Erectile dysfunction after TUMT/TUNA/REZUM/TURP/SHAM Table S2. Comparison of Catheterization and re‐catheterization outcomes after TUNA, TUMT, REZUM studies. Table S3. PSA levels at baseline. The remaining studies have not reported PSA levels at any time point. Table S4. Studies describing the Prostate Volumes at baseline and 3–6 months. Table S5. The different types of devices and techniques used may account for heterogeneous peri‐operative outcomes and their inclusion and exclusion criteria. [file BCO2-5-621-s002.docx]

**Supplementary material**

PubMED/MEDLINE search strategy: ("Prostatic Hyperplasia"[Mesh] OR "Urinary Bladder Neck Obstruction"[Mesh] OR "Lower Urinary Tract Symptoms"[Mesh] "enlarged prostate"[All Fields] OR "benign prostat*" OR "prostatic adenoma") AND (("heat ablat*"[All Fields] OR "transurethral needle ablation"[All Fields] OR "TUNA"[All Fields]) OR ("transurethral microwave thermotherapy"[All Fields] OR "Prostatron"[All Fields] OR "microwave"[All Fields] OR "thermotherapy"[All Fields] OR "thermoablation"[All Fields] OR "TUMT"[All Fields]) OR ("REZUM"[All Fields] OR "water vapour therapy"[All Fields] OR "water vapour thermal therapy"[All Fields]))) OR ("heat ablat*"[All Fields])).

**Supplementary Table 1:** Studies reporting Erectile dysfunction after TUMT/TUNA/REZUM/TURP/SHAM

| **T 1** | **T 2** | **T 3** | **Author** | **Sample Size T 1** | **ED after  T 1** | **Sample Size T 2** | **ED after T 2** |
| --- | --- | --- | --- | --- | --- | --- | --- |
| TUMT | TURP | - | Dahlstrand et al. | 37 | 0 | 32 | 0 |
| TUMT | TURP | - | Norby et al. | 22 | 2 | 7 | 1 |
| TUMT | TURP | - | Wagrell et al. | 80 | 6 | 39 | 6 |
| TUNA | TURP | - | Bruskewitz et al. | 65 | 0 | 56 | 7 |
| TUNA | TURP | - | Cimentepe et al. | 26 | 0 | 33 | 4 |
| REZUM | Sham | - | McVary et al. | 136 | 0** | 65 | 0** |
| TUNA | TUMT | TURP | Arai et al.* | NR | 3.1 | 2.4 | NR |
| TUMT | SHAM | - | Roehrborn et al. | NR | 3 | 2.5 | NR |
| TUNA | NA | - | Murai et al. | 98 | 0 | NA | NA |
| TUNA | NA | - | Campo et al. | 120 | 0 | NA | NA |
| TUNA | NA | - | Roehrborn et al. | 130 | 2 | NA | NA |
| TUNA | NA | - | Namiki et al. | 30 | 0 | NA | NA |
| TUNA | NA | - | Daehlin et al. | 26 | 0 | NA | NA |

ED: Erectile dysfunction; NA: Not applicable; NR: Not reported; T: Treatment; TUMT: Transurethral microwave therapy; TUNA: Transurethral needle ablation; TURP: Transurethral resection of the prostate.
There is significant heterogeneity in the outcomes. *Arai did not report ED outcomes with TURP. **No change in ED from baseline

**Supplementary Table 2:** Comparison of Catheterization and re-catheterization outcomes after TUNA, TUMT, REZUM studies.

| **Study name** | **Catheterization duration (days)** Experimental | **Catheterization duration (days)** Control | **AUR with catheterization** Experimental | **Experimental Group (n)** | **AUR with catheterization after Control** | **Control Group (n)** |
| --- | --- | --- | --- | --- | --- | --- |
| Blute et al.  (TUMT v SHAM) | NR | NR | 20 (25.64%) | 78 | 0 (0%) | 37 |
| Nawrocki et al.  (TUMT v SHAM) | NR | NR | 4 (10.52%) | 38 | 0 (0%) | 40 |
| D'Ancona et al.  (TUMT v SHAM) | 12.7 (6-35) | 4.1 (4-5) | NR | 31 | NR | 21 |
| Francisca et al.  (TUMT v TURP) | NR | NR | 14 | 68 | 30 | 70 |
| Norby et al.  (TUMT v TURP) | 7 prostraton v2.0; 14 prostraton 2.5 [Median] | 2 (1-3) | 3 (6.5%) | 46 | 1 | 22 |
| Cimentepe et al.  (TUNA v TURP) | 0.5-1 | 45325 | 1 (3.84%) | 26 | 0 (0%) | 33 |
| Mcvary et al.  (REZUM v SHAM) | 3.4 ± 3.2 | 0.9 ± 0.8 | 5 (3.67%) | 136 | 0 (0%) | 61 |
| Abbou et al.  (TUMT v SHAM) | NR | NR | 1 (1.51%) | 66 | 0 (0%) | 69 |
| Ahmed et al.  (TUMT v TURP) | NR | 28 | 3 (10%) | 30 | 2 | 30 |
| Albala et al.  (TUMT v SHAM) | 2-4 | 0 | 20 (16.53%) | 121 | 0 (0%) | 62 |
| Bdesha et al.  (TUMT v SHAM) | NR | NR | NR | NR | NR | NR |
| Larson et al.  (TUMT v SHAM) | 1.5-2.5 | 1.5-2.5 | -100% | 125 | 0 (0%) | 44 |
| Norby et al.  (TUMT v TURP) | 14 (1–98) | 2 (1–3) | 26 | NR | 0 (0%) | NR |
| Arai et al.  (TUNA v TURP v TUMT) | NA | NA | NA | NA | NA |  |
| Roehrborn et al.  (TUMT v SHAM) | 2-5 | none | 8 | 147 | 0 (0%) | 73 |
| Murai et al. (TUNA) | 1-7 | NA | NR | 93 | NA | NA |
| Campo B et al. (TUNA) | <2 | NA | NA | 120 | NA | NA |
| Namiki K et al. (TUNA) | NR | NA | NR | 33 | NA | NA |
| Schatzl et al. (TUNA) | 3 | NA | NR | 15 | NA | NA |
| Daehlin et al. (TUNA) | 6.3 (1-34) | NA | NR | 26 | NR | NA |
| Elterman et al (REZUM) | 9 ± 1.75 | NA | 12 | 83 | NA | NA |

Institutional guidelines for each procedure are the main determinant for the length of procedure. Most recatheterizations were performed due to urinary retention. Experimental Group: TUNA/TUMT/REZUM. Control Group: SHAM/TURP.
NA: Not applicable; NR: Not reported; TUMT: Transurethral microwave therapy; TUNA: Transurethral needle ablation; TURP: Transurethral resection of the prostate.

**Supplementary Table 3**: PSA levels at baseline. The remaining studies have not reported PSA levels at any time point.

Most studies used PSA as a cut-off for inclusion and exclusion screening

| **Study** | **Number of patients in Experimental Group** | **Number of patients in Control Group** | **PSA**  **Experimental Group** | **PSA**  **Control Group** |
| --- | --- | --- | --- | --- |
| Blute et al. | 76 | 38 | 3.3 ± 2.9 | 2.9 ± 2.3 |
| Norby et al. | 76 | 38 | 3.3 (1.4-5.7) * | 2.2 (1.5-4.1) * |
| Schelin et al. | 60 | 60 | 7.7 | 6 |
| McVary et al. | 134 | 64 | 2.1 ± 1.5 | 2 ± 1.6 |
| Abbou et al. | 66 | 31 | 4.5 ± 2.7 | 4.2 ± 3 |
| Albala et al. | 121 | 62 | 2.6 ± 1.8 | 2.6 ± 2.3 |
| Larson et al. | 125 | 44 | 3.4 (2.7–4.1) | 3.6 (2.2–5.1) |
| Roehrborn et al. | 147 | 73 | 3.1 ± 2.7 | 2.8 ± 2 |

*Mean with 95% CI

Experimental Group: TUNA/TUMT/REZUM. Control Group: TURP/SHAM

**Supplementary Table 4**: Studies describing the Prostate Volumes at baseline and 3-6 months.

| **Study** | **Sample size Experimental Group** | **Baseline PV Experimental Group** | **Sample size Control Group** | **Baseline PV Experimental Group** | **3–6-month PV Experimental Group** | **3–6-month PV Control Group** |
| --- | --- | --- | --- | --- | --- | --- |
| Dahlstrand et al. | 37 | 33.9 ± 11.9 | 32 | 36.8 ± 16 | NR | NR |
| Nawrocki et al. | 40 | 41.2 ± 14.6 | 40 | 46.7 ± 16.8 | 45.6 ± 17.6 | 48.9 ±19.7 |
| Francisca et al. | 68 | 50 ± 19.4 | 70 | 52 ± 19.2 | 41 ± 16.1 | 23 ± 7.4 |
| Norby et al. | 46 | 43 (35-79) * | 24 | 44 (35-50) * | 37 | 26 |
| Wagrell et al. | 99 | 49 ± 16 | 46 | 53 ± 17 | 34 ± 16 | NR |
| Schelin et al. | 60 | 71.6 | 60 | 66.8 | 56 | 28 |
| Bruskewitz et al. | 65 | 36.2 ± 1.5 | 56 | 35.7 ± 1.9 | 34.4 ± 2.4 | 27.9 ± 2.3 |
| McVary et al. | 136 | 48.8 ±13 | 61 | 44.5 ± 13.3 | NR | NR |
| Ahmed et al. | 30 | 36.6  (31.8–41.4)* | 30 | 46.1  (38.1–54.1) * | 34.5  (29.7–39.3)* | 25.4  (19.4–31.4)* |
| Larson et al. | 125 | 38.1  (35.1–41.2)* | 44 | 44.7  (38.8–50.5)* | - | - |
| Arai et al. | 39 | 10.3 | 27 | 38.6 ± 15.1 | 47 ± 22.1 | NA |
| Roehrborn et al. | 60 | 48.1 ± 16.2 | 60 | 50.5 ± 18.1 | 44.3 ± 20.5 | 46.6 ± 17.8 |
| Murai et al. | 93 | 38.6 ± 15.3 | NA | NR | 29.1 ± 14.2 | NA |
| Namiki et al. | 30 | 37.8 ± 15 | NA | NR | 28.9 ± 12.5 | NA |

Supplementary table 4: most studies chose prostate volumes between 20 – 80 g. *Median (IQR); PV: Prostate Volume
Experimental Group: TUNA/TUMT/REZUM

Control Group: TURP/SHAM

**Supplementary Table 5:** The different types of devices and techniques used may account for heterogeneous peri-operative outcomes and their inclusion and exclusion criteria.

| **Procedure** | **Author** | **Device** | **Inclusion criteria** | **Exclusion criteria** |
| --- | --- | --- | --- | --- |
| TUMT v. TURP | Dahlstrand et al. | Prostatron (Technomed International, France) | Prostatic urethral length 35-50 mm  Madsen and Iverson score ≥ 8 | Indwelling catheter present or PVR >350 mL or Neurogenic bladder disorder  Malignancy of prostate or bladder  Urethral stricture, Large median lobe or Prior BPH treatment  Metallic hip implant present  Previous surgery for pelvic malignancy or regional arterial insufficiency |
| TUMT v. SHAM | Blute et al. | Prostatron treatment (Urologix Inc., USA) | Madsen symptom score ≥ 8  PVR: 100 to 200 mL  Two Qmax <10mL/sec  Prostate length between 35 to 50mm | Concurrent pharmacological therapy  Metallic implants  Neuropathological bladder or asymmetric median lobe enlargement  Prostate cancer or Previous transurethral or rectal surgery  Antiandrogen therapy  Serum Cr >2 mg/dL, urinary retention, bladder stones, uncontrolled dysrhythmias, or cardiac pacemakers |
| TUMT v. SHAM | Nawrocki et al. | Prostasoft v. 2.0 (Prostasoft Inc., USA) | Symptoms of lower urinary tract dysfunction, thought to be due to benign.  enlargement of the prostate meriting surgical treatment  Qmax < 15 mL/s and voided volume <150 mL  Maximum detrusor pressure ≥ 70 cm H_2_O | Complications of bladder outlet obstruction: Urinary retention; PVR >350 mL; Renal failure; Recurrent UTI; Bladder calculus; Bladder diverticulum  Suspicious DRE; Abnormal PSA level  Prostate length < 30 mm  Prominent middle lobe or urethral stricture  Previous prostate or pelvic surgery or radiotherapy  Presence of metal within the lower trunk or upper legs  Uncontrolled cardiac dysrhythmias or presence of a cardiac pacemaker  Presence of neurological disorders that might affect the lower body  Treatment/medication which might affect lower urinary tract function |
| TUMT v. TURP | Francisca et al. | Prostasoft 2.5 (Prostasoft Inc., USA) | Age > 45 years  PV ≥ 30 g  Prostatic urethral length ≥ 25 mm  Madsen symptom score ≥ 8  Qmax ≤ 15 mL/sec  PVR ≤ 350 mL | Acute prostatitis or UTI, or PCa  Isolated obstructed prostatic middle lobe or Intravesical pathology  Diabetes mellitus or Neurological disorders  Current treatment with drugs that may influence the bladder function |
| TUMT v. TURP | Wagrell et al. | ProstaLund, Lund, Sweden | Symptomatic BPH  PV 30-100 g  IPSS ≥ 13  Qmax ≤ 13 mL/sec | NR |
| TUNA v. TURP | Bruskewitz et al. | NR | IPSS ≥ 12  Qmax ≤ 12 mL/sec  Voided volume ≥ 125 mL  PV 20-100 g | Active UTI or Urinary retention  Compromised renal function  Abnormal DRE or Significant median lobe  PSA ≥ 10 ng/mL or PCa  Current use of therapy that affects prostate physiology  PVR ≥ 350 mL |
| TUNA v. TURP | Cimentepe et al. | TUNA system (Vidamed® Inc.) | Age > 40 years  Q_max_ < 15 mL/sec  IPSS > 13  PV 20–70 g  No suspicion of PCa on DRE or PSA | Urethral stricture or BNC  Previous prostate surgery  Bladder stones/tumours or Neurogenic bladder  Prominent median lobe |
| REZUM v. SHAM | McVary et al. | Rezūm System (NxThera, Inc., Maple Grove, MN) | Male ≥ 50 years of age who have symptomatic BPH  IPSS ≥ 13  Qmax 5-15 mL/s  PV 30-80 g  Washout period: antihistamines (1 week); α-blockers, anticholinergics, or daily PDE-5 inhibitors (4 weeks); estrogen/androgen suppressing drugs, anabolic steroid, or Type II 5αRI (3 months); dual 5αRI (6 months) | PVR ≥ 250 mL  PSA ≥ 2.5 ng/ml with a free PSA ≤ 25% (unless prostate cancer was ruled by biopsy)  Active UTI within 7 days or two independent infections within the last 6 months  Subjects with median lobes not excluded  Previous prostate procedure |
| TUMT v. SHAM | Abbou et al. | Thermex II, (Technorex Inc., Israel)  Prostcare, Brucker Spectrospin, France; BSD-50. BSD Medical Corp, USA (three instruments were used) | Age > 50 years  PV 30-80 g  Qmax < 15 mL/s  PVR < 300mL  PSA < 10ng/mL (PV < 60g) or PSA < 15ng/mL (PV ≥ 60g)  Serum creatinine level < 160 µmol/L; | Previous surgery on the prostate or bladder  mental incapacity or diabetes  Any other urological disease  Medical treatment for voiding disorders within 15 days of inclusion  Use of diuretics in the previous 3 months.  anticoagulant therapy, allergy to lidocaine, or colorectal disease |
| TUMT v. TURP | Ahmed et al. | Prostatron™ version 2.5 (Urologix Inc., USA) | AUA score ≥12  Qmax < 15 mL/sec  PVR < 300 mL  Pdet max ≥70 cm H_2_O  PV 25–100 g  Obstructed as assessed on the Abrams-Griffith nomogram  Aged ≥ 55 years | Mental incapacity, severe CV disease, ‘active’ drugs  Metallic implants; Cardiac pacemaker; Rectal surgery/disease (except haemorrhoids); Pelvic mass/surgery; Previous prostatic surgery; Prostatic abscess; Uncontrolled coagulation disorder; Active UTI  Prominent middle lobe; Meatal stricture Previous drug treatment for BPH  Acute or chronic urinary retention; Upper tract dilatation; Obstructive uropathy (serum Cr>150 mmol/L); Bladder calculi; Bladder diverticula; Recurrent UTI; Recurrent prostatic; haematuria |
| TUMT v. SHAM | Albala et al. | TherMatrx TMx-2000, (TherMatrx, Inc., USA) | AUASI > 13 and bother score >11  PFR<12 mL/sec,  PVR < 125 mL  PV 30-100 g without a significant intravesical middle lobe | NR |
| TUMT v. SHAM | Bdesha et al. | LEO Microthermer (Laser Electro Optics Ltd., UK) | symptomatic for least 6 months in duration  symptom score ≥10  Qmax ≤ 15 mL/sec  PVR ≥ 50 mL | upper tract dilatation, impaired renal function, or AUR  PVR ≥200 mL  PCa or previous prostatic surgery  Middle lobe or bladder neck to mid-verumontanum distance ≥40mm  coexisting urinary tract pathological condition |
| TUMT v. SHAM | Larson et al. | Targis transurethral thermoablation system – (Urologix Inc, USA) | Age: 45-85 years  Qmax≤ 12mL/sec  AUA symptom score ≥9 3-5 cm prostatic urethral length  No disproportionately enlarged prostatic median lobe  Life expectancy ≥ 1 year | UTI within 1 week of enrolment  Gross haematuria  AUR or PV >100g  Use of α-antagonists (4 weeks) or antiandrogens (3 months) of enrolment  Neurogenic bladder/bladder stones, renal or cardiac failure, PCa, urethral stricture, BNC, urinary sphincter abnormalities, prostatitis, hepatic failure  Urinary catheterisation within 2 weeks of study  Previous prostate procedure ≤12 months of the study  Penile implant or artificial urinary sphincter or Metallic implant in pelvic area  Previous pelvic or rectal surgery  Cardiac pacemaker  Desire for offspring |
| TUMT v. TURP | Norby et al. | TUMT- Prostatron system, (Urologix, USA)  TURP – Iglesias resectoscope | Age ≥ 50 years  IPSS ≥7  QoL ≥3  Qmax <12mL/sec | Suspicion of PCa, previous prostatic surgery or pelvic radiation therapy  PVR >350 mL or urinary catheter  Prostatic urethra < 25mm  Neurological disease or diabetes with abnormal cystometry  Ongoing UTI or medications known to influence voiding  Rectal cancer  Severe peripheral arterial disease |
| TUMT v. SHAM | Venn et al. | Machine constructed with Microwave Engineering Designs, UK | Madsen score >8  PVR <250 mL  Predominantly lateral lobe enlargement  No evidence of prostate or bladder cancer  No previous lower urinary tract surgery | NR |
| TUMT v. TUNA v. TURP | Arai et al. | TURP – Storz resectoscope; TUMT – Dornier Urowave (Dornier Inc., USA)  TUNA – NR | NR | NR |
| TUMT v. SHAM | Roehrborn et al. | Dornier Urowave (Dornier Inc., USA) | Age ≥ 55 years  AUA-SI ≥13  Qmax ≤ 12mL/sec  Serum PSA < 10 ng/mL  PV 25-100 g  Bladder neck to verumontanum ≥30 mm | (reported as inclusion and exclusion criteria) |
| TUNA | Murai et al. | NR | Age ≥ 50 years  IPSS ≥13  Qmax ≤ 10mL/sec  PVR <250mL  PV: 15 to 75 g | PCa, urothelial malignancy, bladder stones, prostatitis, prostatodynia, neurogenic bladder, urethral stricture, pelvic surgery/injury  Serum PSA level >10 ng/mL, serum creatinine > 1.8mg/dL, blood coagulation disorders, use of anticoagulants, hydronephrosis  Diabetes mellitus |
| TUNA | Campo et al. | TUNA system | IPSS ≥13  PV 15-75 g  Age: 50-75 years  Qmax ≤ 13mL/sec  Lateral lobe hyperplasia only | Neurological or psychiatric disorder  Coagulopathy or Gross haematuria  Urethral strictures |
| TUNA | Roehrborn et al. | TUNA system | IPSS ≥ 13  PV: 20-75 g  Qmax ≤ 12mL/s  PVR ≥ 125 mL | PVR > 350 mL  Serum PSA ≥10 ng/mL  Prostate malignancy |
| TUNA | Namiki et al. | TUNA system | Age ≥ 50 years  IPSS ≥ 13  Qmax ≤ 12 mL/sec on voided volume of ≥ 150 cc  PV: 15- 75 g | Neurogenic bladder and/or sphincter abnormalities  Active cystolithiasis, hematuria, urethral stricture, BNC  Active UTI, Compromised renal function or urinary tract obstructive disease  PSA ≥ 10 ng/mL or previous prostatic surgery  Confirmed or suspected malignancy of the prostate or bladder |
| TUNA | Zlotta et al. | NR | NR | PV > 90 g  Previous prostate surgery, PCa, urethral stricture, BNC, or neurogenic bladder |
| TUNA v. TURP | Schatzl et al. | NR | Qmax ≤ 15mL/sec  IPSS ≥ 7  Linear passive urethral resistance relation ≥ 2 | Previous prostatic surgery, bladder neck, urethra  Neurogenic bladder or Urinary retention  PV > 75 g or Middle lobe exceeding 1.5cm |
| TUNA | Daehlin et al. | NR | Age ≥ 45 years  IPSS > 10  Qmax ≤ 15mL/sec | Recurrent UTI  Urinary retention/indwelling urinary catheter or Previous prostate surgery  PCa or Serum PSA > 20µg/L, Serum creatinine >150 µmol/L  PVR >300 mL  Medication for LUTS  Previous pelvic radiation, neurological disorders involving bladder  Infravesical obstruction not due to BPH |
