# Supplementary material for: A systematic review and network meta‐analysis comparing Rezūm with transurethral needle ablation and microwave thermotherapy for the management of enlarged prostate
Source: BJUI Compass. 2024 Apr 29;5(7):621–35. doi: 10.1002/bco2.361 (PMC11250421; doi:10.1002/bco2.361)

**Supplementary Figure 1a**: Funnel plot for assessing publications bias in IPSS at 3 months.


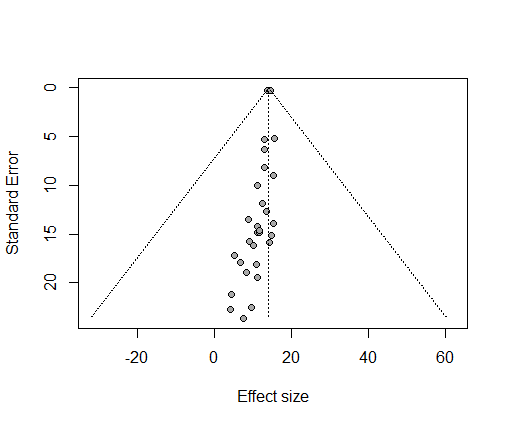


**Supplementary Figure 1b:** Funnel plot for assessing publications bias in IPSS at 12 months.
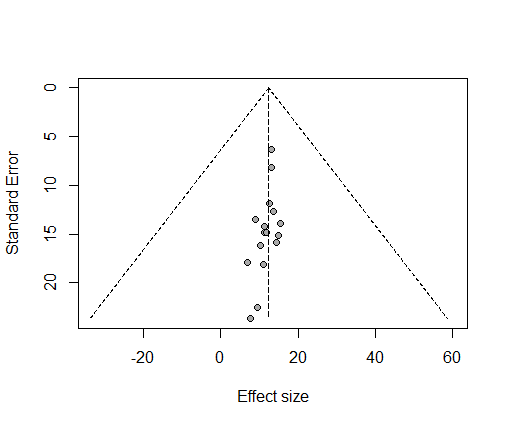


**Supplementary Figure 1c:** Funnel plot for assessing publication bias in Qmax at 3 months.


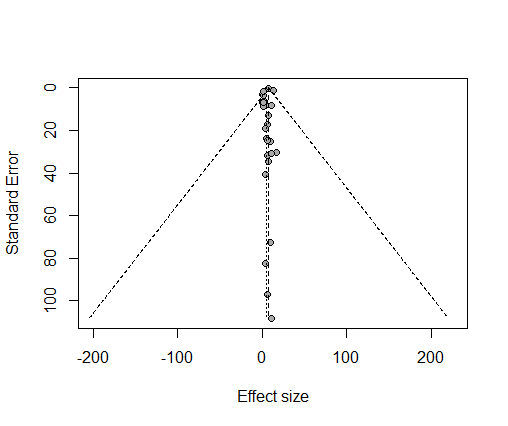


**Supplementary Figure 1d:** Funnel plot for assessing publication bias in Qmax at 12 months.


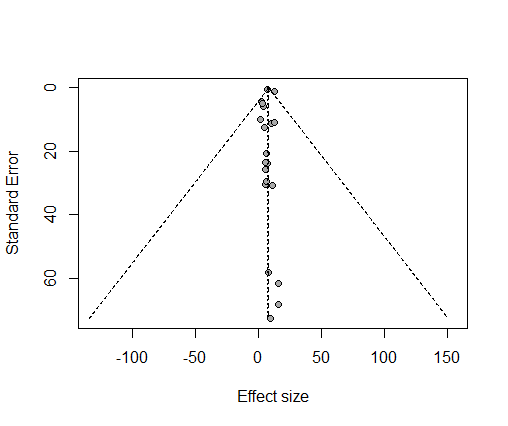


**Supplementary Figure 1e:** Funnel plot for assessing publication bias in retreatment rate analysis.


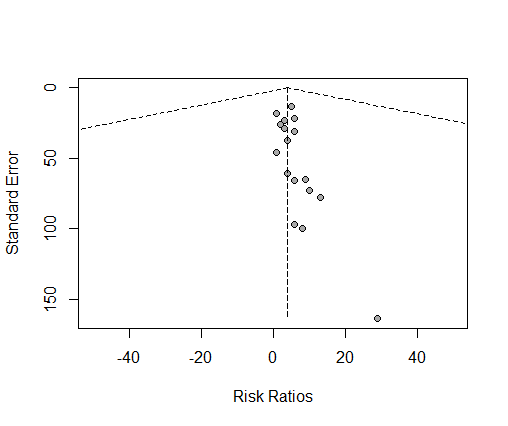


**Supplementary Figure 1f:** Funnel plot for assessing publication bias in serious adverse event analysis.


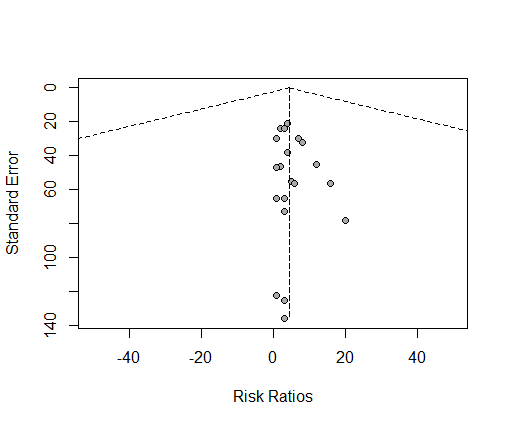

Supplement: Supplementary file 2 — Figure S1. Funnel plot for assessing publications bias in IPSS at 3 months. [file BCO2-5-621-s001.docx]
